# Blood-based biomarkers in mild behavioral impairment: an updated overview

**DOI:** 10.3389/fneur.2025.1534193

**Published:** 2025-02-06

**Authors:** Efthalia Angelopoulou, Xenia Androni, Chiara Villa, Alexandros Hatzimanolis, Nikolaos Scarmeas, Sokratis Papageorgiou

**Affiliations:** ^1^1st Department of Neurology, Aiginiteio University Hospital, National and Kapodistrian University of Athens, Athens, Greece; ^2^School of Medicine and Surgery, University of Milano-Bicocca, Monza, Italy; ^3^1st Department of Psychiatry, Aiginiteio University Hospital, National and Kapodistrian University of Athens, Athens, Greece

**Keywords:** Mild behavioral impairment, biomarkers, Alzheimer’s disease, dementia, neuropsychiatric symptoms

## Abstract

Identifying individuals at-risk for dementia is one of the critical objectives of current research efforts, highlighting the need for simple, cost-effective, and minimally invasive biomarkers. Mild behavioral impairment (MBI), characterized by the emergence of persistent neuropsychiatric manifestations in older adults, has attracted increasing attention as a potential early indicator of cognitive decline and dementia. A growing number of studies have recently begun to explore the relationship between MBI and several blood-based biomarkers associated with Alzheimer’s disease (AD) pathology, neurodegeneration, as well as systemic metabolic and inflammatory dysregulation. In this context, MBI has been associated with lower plasma Aβ42/Αβ40 ratio, higher plasma phosphorylated tau at threonine 181 (p-tau181), increased neurofilament light chain (NfL) levels, as well as disturbances in metabolic markers, including homocysteine, insulin and ferritin, suggesting a multifaceted neurobiological basis for this syndrome. These findings offer insights into the underlying pathophysiology of MBI, and connection between neuropsychiatric symptoms and progression of AD. In this narrative review, we aim to summarize and critically discuss the emerging literature evidence linking MBI to blood-based biomarkers, hoping to shed more light on MBI’s pathophysiology, its connection to AD-related neurobiology, as well as its potential practical utility for predicting cognitive impairment, guiding early interventions and managing the risk for dementia.

## Introduction

Dementia is a clinical syndrome characterized by cognitive decline leading to functional impairment in the activities of daily living (1, 2). Due to the aging population and increased longevity, the prevalence of dementia will continue to rise, resulting in a growing health, economic and social burden worldwide. Currently, about 50 million people suffer from dementia, while this number is estimated to triple by 2050 (1). Mild cognitive impairment (MCI) is characterized by objective deficits in cognitive performance, while the patient maintains functional independence. Individuals with subjective cognitive decline (SCD) display cognitive complaints, with no objective impairment in neuropsychological testing (1). SCD, MCI and dementia are considered the main parts of the neurocognitive axis of the continuum of cognitive decline (1).

Alzheimer’s disease (AD) is the leading cause of dementia, representing about 65% of all cases (3). Clinically, patients with AD exhibit progressive deficits in cognitive domains, including short-term memory, language, executive function, attention and visuospatial abilities, as well as neuropsychiatric symptoms (NPS), including anxiety, depression, apathy, agitation, irritability and impaired social cognition (3). The neuropathological hallmarks of AD are the extracellular deposition of amyloid plaques and the intracellular accumulation of neurofibrillary tangles of tau protein (3). Importantly, these neuropathological changes may be evident even two decades before the onset of memory impairment (4). However, by the time dementia is clinically diagnosed, the AD-related neuropathological changes and neuronal loss are typically widespread and likely irreversible.

There is no globally approved disease-modifying therapeutic agent against AD, able to halt disease progression. Identifying AD at the earlier phases is required for the discovery of effective novel treatments, ideally before the onset of cognitive decline (3). Clinical trials are often characterized by low recruitment rates of participants, particularly in the early stages of the disease, and this limitation is considered one of the main reasons for their failure (5). In this context, there is a pressing need for the development of efficient approaches to identify individuals at an elevated risk of incident dementia.

The neuropathological changes of AD are reflected in its biochemical profile in both fluid and neuroimaging biomarkers, characterized by reduced amyloid-beta (Aβ) levels and elevated concentrations of total and phosphorylated tau protein (3). Cerebrospinal fluid (CSF) amyloid and tau levels, as well as amyloid and tau positron emission tomography (PET), have been widely used as validated biomarkers for the early detection of AD (6). However, the lumbar puncture needed for the CSF examination is an invasive process, and it cannot be easily performed especially in remote, undeserved areas (6). Amyloid and tau PET scans are also quite expensive, and unavailable in many areas of the world, limiting the feasibility of these approaches on a population-wide scale (6). Therefore, simple, minimally invasive, cost-effective and highly accessible alternative methods are required.

Blood-based biomarkers represent an attractive approach for this emerging need. They constitute a feasible, minimally invasive, and relatively inexpensive tool, serving as potential biomarkers of the underlying neuropathology (6). Blood testing requires a quite simple equipment and infrastructure and constitutes a well-established process of clinical practice, necessitating no additional training for healthcare professionals (7). Blood testing can be also conducted in various healthcare settings even in patients’ homes, and blood samples can be quite easily shipped from primary care settings to appropriate laboratories, enabling their use as an effective tool at initial screening stages (7). Finally, the use of blood samples allows for a comprehensive analysis of a wide range of underlying pathophysiological mechanisms, beyond the traditional biomarkers related to amyloid or tau pathology (7).

The plasma Aβ42/Aβ40 ratio is considered an effective early marker of the brain amyloid pathology (8). Plasma p-tau181 is another key biomarker of AD-related tau pathology, which is also associated with disease progression and more rapid cognitive decline (9). Blood neurofilament light chain (NfL) is a sensitive biomarker of axonal injury and neurodegeneration, whose levels are increased in AD and other neurological disorders (10). These biomarkers can be detected in early stages of AD, even before the clinical onset of cognitive impairment (10). Given their growing integration into daily clinical practice, elucidating their role in preclinical and prodromal dementia states is essential for their optimal use.

Although NPS are generally considered core manifestations of advanced stages of AD dementia, their occurrence may actually precede the onset of cognitive impairment. While about 10–15% of patients with MCI develop dementia each year, this percentage increases to 25% in the presence of NPS (11). According to the National Institute of Aging-Alzheimer’s Association (NIA-AA) Framework, stages 1 and 2 constitute the preclinical phase of the AD continuum, while stage 3 represents the prodromal phase of the disease (12). In stage 1, the individual is asymptomatic, with objectively normal cognitive performance. In stage 2, there is subtle impairment or subjective complaints. In stage 3, impaired cognition is objectively observed, but the individual remains functionally independent. As stated in the NIA-AA Framework, although in stages 2 and 3 the core clinical characteristic are cognitive deficits, the main concerns may actually be behavioral changes, which may offer a valuable opportunity for detection and intervention at early stages.

Mild behavioral impairment (MBI) is a validated clinical syndrome, characterized by the emergence of persistent NPS in non-demented older adults, which may indicate a higher risk for cognitive decline (13). According to the NPS Professional Interest Area (PIA) of the Alzheimer’s Association (AA) subgroup International Society to Advance Alzheimer’s Research and Treatment (ISTAART), for the MBI diagnosis, NPS should persist for at least 6 months, and not be attributed to a pre-existing psychiatric or other medical condition (13). The criterion of 6-months persistence aims to increase the likelihood that these NPS are not reactive to transient life events but a part of the neurodegenerative process (13). According to the ISTAART-AA MBI Criteria, NPS belong to five domains: decreased motivation (e.g., apathy, indifference, aspontaneity), affective dysregulation (e.g., depressive symptoms, anxiety, dysphoria, changeability, euphoria and irritability), social inappropriateness (e.g., loss of insight, lack of empathy, loss of social graces or tact, rigidity, exaggeration of previous personality traits), abnormal perception or thought content (e.g., hallucinations, delusions), and impulse dyscontrol (e.g., impulsivity, aggression, gambling, disinhibition, agitation, obsessiveness, behavioral perseveration, stimulus bind) (13). Although the individual should be functionally independent, these NPS should create some impact on the professional activities, social life or interpersonal relationships (13). MBI might occur even before the onset of cognitive complaints, and it can co-exist with MCI (13). MBI can be identified using the validated MBI-Checklist (MBI-C), which has been constructed specifically for capturing MBI, or a published algorithm based on the Neuropsychiatric Inventory (NPI) and Neuropsychiatric Inventory Questionnaire (NPI-Q) (13, 14). NPI, NPI-Q and MBI-C can be relatively easily performed, representing inexpensive and non-invasive tools for evaluating the behavioral symptoms of pre-dementia at-risk states (13, 14).

Since the MBI construct is relatively new, investigating its relationship with well-established biomarkers of AD and other causes of cognitive impairment is required. Investigating the neurobiological correlates of MBI will aid in the elucidation of its underlying pathophysiological mechanisms, its utility in the daily clinical practice, as well as the design of clinical trials (15). MBI has been associated with amyloid and tau pathology in studies using PET and CSF, grey matter atrophy, and genetic risk factors for AD (15, 16). However, the association between MBI and blood-based biomarkers has only recently started to be explored. MBI and blood-based biomarkers including amyloid beta, tau and NfL have been independently implicated in neurodegeneration and incident dementia (17–20). Understanding the associations between MBI and biomarkers of neurodegeneration will contribute to more accurate risk stratification, allowing for monitoring disease progression, and enabling appropriate intervention at the preclinical and prodromal stages of cognitive decline.

The role of genetic factors and potential underlying pathophysiology of MBI, as well as general overviews of this entity have been already reviewed elsewhere (15, 16, 21). However, there is no recent overview focusing on the relationship between blood-based biomarkers and MBI. Given the increasing research attention and growing body of literature evidence in this field, in this narrative review, we aim to explore the relationship between blood-based biomarkers and MBI, hoping to shed more light on our understanding of its underlying neurobiology and clinical implications. Finally, we provide future perspectives that might help toward the development of targeted interventions at the early stages of neurodegeneration and cognitive impairment in the context of MBI.

## Methods

Despite the narrative nature of our review, we followed a systematic approach. We searched MEDLINE and Scopus databases for clinical studies exploring the association between blood-based biomarkers and MBI published in English without time restrictions. Our search was conducted between August 2024 and October 2024. We used the terms “mild behavioral impairment,” “neuropsychiatric symptoms,” “affective dysregulation,” “decreased motivation,” “social inappropriateness,” “impulse dyscontrol,” “abnormal perception or thought content,” “anxiety,” “depression,” “apathy,” “psychosis,” “delusions,” “hallucinations,” “agitation,” “aggression,” “mild cognitive impairment,” “subjective cognitive decline,” “Alzheimer’s disease,” “biomarker,” “blood,” “blood-based,” “serum,” “plasma,” “amyloid” and “tau” in different combinations. After screening the title and abstract, we read the full forms of the relevant studies. The bibliography of each relevant article was also screened for additional articles. We primarily included studies investigating various blood-based biomarkers related to MBI. For discussion purposes, we also referred to clinical studies or review articles exploring NPS not defined as MBI, NPS in dementia, MBI or NPS in other neurodegenerative diseases, as well as *in vivo* and *in vitro* studies for the possible underlying pathophysiological mechanisms.

We defined the different MBI domains according to the ISTAART-AA MBI Criteria: (1) decreased motivation, (2) affective dysregulation, (3) impulse dyscontrol, (4) social inappropriateness and (5) abnormal perception or thought content. We included studies using either MBI-C, NPI or NPI-Q for MBI assessment, based on a previously published transformational algorithm, which maps the 12 NPI and NPI-Q items onto the 5 MBI-C domains (13, 14). According to this approach, the NPI and NPI-Q neurovegetative items K and L (night-time behaviors, appetite and eating) are excluded, since they are not reflected on the MBI criteria. To meet the operationalized criteria for a specific MBI domain, participants are required to exhibit at least one NPI symptom corresponding to that domain, as follows: to fulfill the criteria for affective dysregulation, participants need to present with either depression/dysphoria, anxiety, or elation/euphoria, as assessed by the NPI items D, E and F accordingly. For decreased motivation, participants need to present with apathy/indifference, as assessed by the NPI item G. For impulse dyscontrol, participants need to present with either agitation/aggression, irritability/lability, or aberrant motor behavior as assessed by the NPI items C, I and J accordingly. For social inappropriateness, participants need to present with disinhibition, as assessed by the NPI item H. For abnormal perception or thought content, participants need to present with either delusions or hallucinations as assessed by the NPI items A and B accordingly. Participants were considered to meet the overall criteria for MBI if at least one of the five MBI domains was present. If no symptoms were present in a given domain, that domain was assigned a score of zero.

## Results

### MBI and blood-based amyloid-beta (Aβ)-related biomarkers

Abnormal amyloid metabolism and amyloid deposition in the form of senile plaques are core features of AD neuropathology (3). According to the “amyloid cascade hypothesis,” increased levels of amyloid beta peptides, and particularly amyloid beta 42, is the key pathogenic process of AD, triggering tau deposition and subsequently neuronal loss (3). Amyloid beta is derived from the cleavage of the amyloid precursor protein (APP), which is widely expressed in neurons (22). The two main enzymes implicated in the proteolysis of APP are *β*-site APP cleaving enzyme-1 (BACE1), also known as β-secretase, and *γ*-secretase (22, 23). Amyloid beta deposition can disrupt synaptic transmission and neurotransmitter signaling, dysregulate calcium homeostasis, promote excessive neuroinflammation and oxidative stress, as well as contribute to blood–brain barrier and vascular dysfunction, thereby leading to neuronal cell death (22, 23). Importantly, amyloid deposition occurs many years before the clinical onset of AD, highlighting its role as a potential biomarker of the early stages of the disease (22, 23) (Table 1).

**Table 1:** A summary of main studies on blood-based biomarkers in mild behavioral impairment.

References	Study type	Study population	MBI assessment	Blood-based biomarker assessment	Main findings
Ghahremani et al. (18) (data based on ADNI)	Longitudinal (4 years)	571 non-demented individualsAge (mean): 72.2 years, 46.8% females201 NC, 370 MCI	NPI or NPI-Q (transformation based on the published algorithm) MBI defined as NPI > 0 in both visits (baseline and year 1); NPS-not-MBI defined as NPI > 0 in 1 visit; No-NPS: NPI = 0 in both visits	Plasma p-tau181 (SIMOA)	Cross-sectionally, MBI (but not NPS-not-MBI) at baseline was related to 8.1% higher levels of p-tau181, compared to no-NPS. Lower MMSE scores were also associated with higher p-tau181 levels. Longitudinally, MBI was related to higher levels of p-tau181, cognitive decline assessed by MMSE over a 4-year period, and impairment in memory and executive function, assessed by RAVLT and Trail Making B test, respectively. Interaction analyses between p-tau181 status and NPS groups indicated that in the p-tau181-positive subgroup, MBI was associated with 2.56 times higher incidence of dementia over the 4-year follow-up period, compared to no-NPS
Miao et al.(17) (data based on ADNI)		139 non-demented individualsAge (mean ± SD): 72.4 ± 7.6 years, 51.8% females86 NC, 53 MCI	NPI (transformation based on the published algorithm)MBI total score based on NPI score; MBI domain scores based on the NPI published algorithm	Plasma Aβ42/Aβ40 ratio	Higher MBI score was related to lower Aβ42/Aβ40 ratio. Affective dysregulation, but neither impulse dyscontrol nor decreased motivation were related to Aβ42/Aβ40 ratio. Cognitive status at baseline (NC versus MCI) was not associated with Aβ42/Aβ40 ratio
Gonzalez-Bautista et al. (19) (data based on the NOLAN study, clinical trial NCT03080675)	Longitudinal (1 year)	359 non-demented older individuals with self-reporting subjective memory complaintsAge (mean ± SD): 78.3 ± 0.3 years, 60% females	NPI-Q (transformation based on the published algorithm)MBI approached in terms of MBI domain status; MBI domain status was defined as positive in case at least one behavior in NPI-Q - comprising a specific MBI domain- was positive; MBI domain severity was defined as the arithmetical addition of the severity from the corresponding NPI-Q items of each MBI domain	Plasma p-tau181 (SIMOA), GFAP (GFAP Discovery assay), *ω* − 3 index (the sum of percentage docosahexaenoic acid and percentage eicosapentaenoic acid, expressed as the percentage of total erythrocyte membrane fatty acids), homocysteine, insulin, 25 hydroxyvitamin D, ferritin, and transferrin	Abnormal perception or thought content (presence and severity) was associated with faster increase in p-tau181 at 1 year follow-up. Abnormal perception or thought content was associated with a more rapid increase in homocysteine levels. Decreased motivation and impulse dyscontrol were associated with faster reduction in insulin levels. No significant associations were observed between MBI and other inflammatory or metabolic biomarkers (GFAP, 25 hydroxyvitamin D, ferritin and transferrin, ω-3 index)
Naude et al. (20) (data based on ADNI)	Longitudinal (2 years)	584 non-demented individuals (ADNI participants with available NfL levels at baseline and at 2-years of follow-up, cognitive status at baseline, and NPI-Q score administered within 6 months of the baseline NfL levels)	NPI-Q (within 6 months of NfL baseline measurement, transformation based on the published algorithm)MBI defined as NPI-Q total score in the 10 MBI domains >1, non-MBI defined as NPI-Q total score in the 10 MBI domains = 0. Participants with NPI-Q total score = 1 were excluded because of diagnostic uncertainty	Plasma NfL levels (SIMOA)	MBI at baseline was associated with faster longitudinal increase in NfL concentration. MBI was the only variable that could significantly predict the change in NfL levels, after controlling for cognitive status, age, sex and years of education. Participants that developed NPS at 2 years demonstrated higher NfL levels at baseline, compared to those who remained without NPS
Leow et al. (73) (data based on the baseline data from BIOCIS)	Cross-sectional	607 non-demented individualsAge (mean ± SD): 61.99 ± 10.19 years, 31.1% males126 (20.8%) NC, 156 (25.7%) SCD, 325 (53.5%) MCI	MBI-C	Fasting total cholesterol and fasting blood glucose	Fasting glucose levels were associated with social inappropriateness

ADNI, Alzheimer’s Disease Neuroimaging Initiative; BIOCIS, Biomarkers and Cognition Study, Singapore; MBI-C, Mild behavioral impairment – checklist; MCI, mild cognitive impairment; MMSE, Mini-Mental State Examination; NC, normal cognition; NfL, Neurofilament light chain; NPI-Q, Neuropsychiatric Inventory Questionnaire; p-tau181, plasma phosphorylated tau at threonine 181; RAVLT, Rey Auditory Verbal Learning Test; SCD, subjective cognitive decline; SD, standard deviation.

Higher amyloid deposition as assessed by amyloid PET and lower amyloid beta 42 levels in the CSF are highly sensitive biomarkers of AD-related amyloid pathology (24). However, their increased cost, limited availability or invasiveness restricts their use at a population-level (24). In this regard, plasma Aβ42/Aβ40 ratio has emerged as a promising biomarker for AD, being able to predict AD neuropathology in at-risk older adults (25). Plasma Aβ42/Aβ40 correlates well with both amyloid PET and CSF amyloid biomarkers of AD in non-demented individuals (26). A decreasing Aβ42/Aβ40 ratio has been linked to progression in early AD (27, 28), highlighting its important value as a screening biomarker in prodromal and preclinical stages (17). Notably, positive plasma Aβ42/Aβ40 in individuals with negative amyloid PET display a higher risk for conversion to amyloid PET positivity (29), implying that plasma Aβ42/Aβ40 might depict amyloidopathy earlier compared to amyloid PET. Aβ42/Aβ40 ratio demonstrates higher diagnostic accuracy compared to Aβ42 or Aβ40 levels (25, 30). While the predictive value of Aβ42 and Aβ40 levels is influenced by age, that of Aβ42/Aβ40 ratio remains unaffected (25). Hence, it seems that plasma Aβ42/Aβ40 ratio represents a useful biomarker of early AD amyloidopathy.

**Figure 1:**
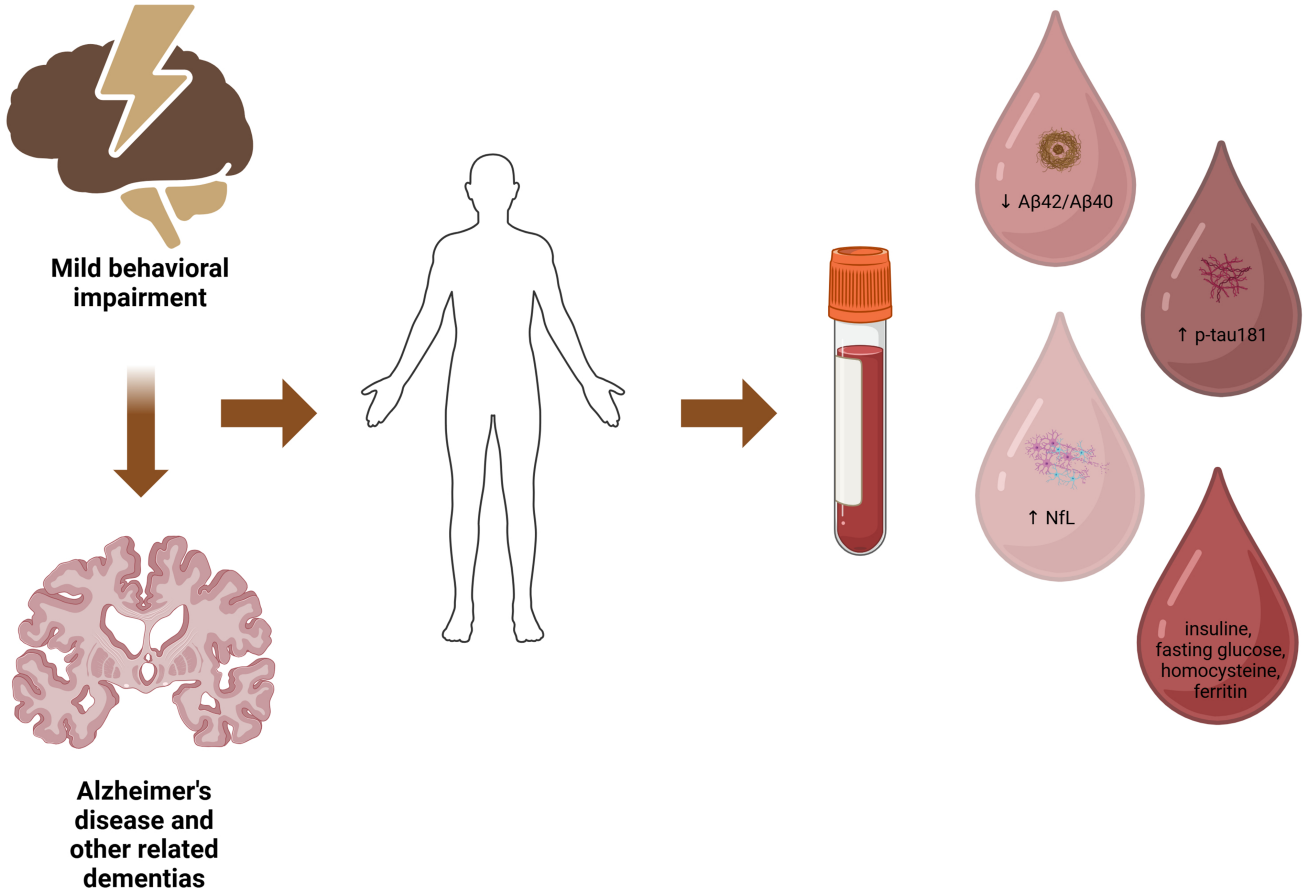
The potential relationship between mild behavioral impairment, blood-based biomarkers and underlying neuropathology. Created in BioRender. Villa (101).

MBI has been associated with lower beta amyloid in CSF (31), as well as increased amyloid deposition in amyloid PET (31) in non-demented older individuals. A recent study by Miao et al. (17) using data from ADNI revealed that a higher MBI total score, as evaluated by NPI, was associated with a lower plasma Aβ42/Aβ40 ratio in non-demented older individuals (Figure 1). On the other hand, cognitive status at baseline (normal cognition versus MCI) was not associated with Aβ42/Aβ40 ratio (17). Concerning the specific MBI domains, in the study by Miao and colleagues, affective dysregulation was the only one associated with decreased Aβ42/Aβ40 ratio (17). Affective dysregulation, mainly including anxiety and depression, has been previously associated with AD biomarkers (32–34). In this context, anxiety symptoms have been correlated with lower Aβ42 levels in the CSF (32–34), and elevated plasma Aβ42 concentration has been associated with a higher risk for a first episode of depression in older individuals with normal cognition (35). Furthermore, deteriorating depressive symptomatology during a period of 2–7 years in older individuals with normal cognition was related to cognitive decline, while this relationship was influenced by amyloid deposition in PET (36). These findings support the value of MBI, and particularly the domain of affective dysregulation, as an early marker of amyloid pathology even independently to cognitive performance, suggesting also that MBI might be a more sensitive indicator of AD neuropathology compared to cognitive decline.

Proposed pathophysiological mechanisms linking amyloid pathology with MBI involve neuroinflammation, neurotrophic factor dysregulation, as well as synaptic dysfunction and neurotransmitter imbalance including glutamate, acetylcholine, noradrenaline, serotonin, dopamine, and gamma-aminobutyric acid (GABA) among others (15).

Collectively, these findings suggest that plasma Aβ42/Aβ40 ratio might be associated with MBI. The combined use of MBI and Aβ42/Aβ40 in patients with SCD or MCI may contribute to more precise risk stratification for AD dementia, and future longitudinal studies using MBI-C are required toward this direction.

### MBI and blood-based tau-related biomarkers

Although the deposition of amyloid beta Aβ is widely known as the core process of the AD-related neurodegeneration, it has been recently indicated that tau accumulation might actually occur before amyloidosis (37). Tau is a microtubule-associated protein that plays an important role in the stabilization of cytoskeleton and axonal transport. Tau pathology has been associated with the degree of neuronal loss and the severity of clinical symptoms (37). Tau-mediated neurotoxicity has been linked to several neurobiological mechanisms, including synaptic loss, mitochondrial dysfunction, and activation of neuroinflammatory pathways (37).

Apart from tau biomarkers in the CSF and PET, plasma phosphorylated tau at threonine 181 (p-tau181) is a validated AD-specific biomarker, demonstrating significant sensitivity for the prediction of incident cognitive decline (38–40). Notably, plasma p-tau181 shows better accuracy in determining the risk for progression to dementia in AD, compared to other plasma biomarkers, including total tau (41), Aβ42/Aβ40 (42) and NfL (43). Plasma p-tau181 can also precisely discriminate AD from other neurodegenerative disorders (44–46). Across the continuum of AD, increased plasma p-tau181 levels have been correlated with amyloid beta and tau pathologies in the brain, as well as brain atrophy (38, 40).

Some NPS in AD dementia, including psychosis, have been associated with tau deposition (47). In mild AD, apathy has also been correlated with higher p-tau181 levels in the CSF (48). MBI has been related to higher levels of phosphorylated tau in the CSF (49), and CSF p-tau181 has been longitudinally associated with higher scores in the NPI-Q in non-demented older individuals (50). MBI has also been associated with higher tau deposition in tau PET (49), although there is also evidence not confirming the relationship (31, 51). Based on this evidence, the relationship between MBI and plasma p-tau181 has been recently investigated.

In particular, a study by Ghahremani et al. (18) in 571 non-demented older individuals based on the data from ADNI, aimed to explore the cross-sectional and longitudinal associations between plasma p-tau181 and MBI, assessed by the NPI or NPI-Q at two follow-up visits over a period of 4 years. Cross-sectionally, MBI was related to higher levels of p-tau181 at baseline, compared to no-NPS. However, no significant relationships were demonstrated between p-tau181 and NPS-not-MBI -defined as those with NPS not fulfilling the persistence criterion (18). Longitudinally, MBI was related to increasing levels of p-tau181, global cognitive decline, as well as decline in memory and executive function over a 4-year period (18). Similarly to the cross-sectional evidence, no significant longitudinal alterations in p-tau181 levels, as well as episodic memory and executive function were observed for the NPS-not-MBI group (18). Notably, interaction analyses between p-tau181 status and NPS groups demonstrated that in the p-tau181-positive subgroup, MBI was related to 2.56 times higher incidence of dementia over the 4-year follow-up period compared to no-NPS (18). These findings imply that even among p-tau181-positive individuals, the emergence of persistent NPS indicates an even higher risk for incident dementia due to AD (18). Collectively, this evidence further confirms the validity of MBI as an effective predictor of cognitive decline and highlights the important criterion of persistence in the assessment of MBI.

In the same context, a recent study by Gonzalez-Bautista et al. (19) based on a secondary analysis of the clinical trial NOLAN aimed to assess the longitudinal relationship between MBI domains and several plasma biomarkers, including p-tau181 in 359 non-demented individuals. In this study, abnormal perception or thought content was associated with faster increase in p-tau181 at 1-year follow-up (19). In addition, supplementary analyses in this study revealed a significant interaction between impulse dyscontrol and Clinical Dementia Rating (CDR) score regarding the relationship between MBI and p-tau181 concentration (19). This finding implies that as cognitive performance worsens, the MBI domain of impulse dyscontrol might become more strongly related to tauopathy.

Although the tau-related mechanisms of MBI remain largely unclear, several mechanisms have been proposed to be involved, including the neurotransmitter and neurotrophic factor imbalance, locus coeruleus-norepinephrine system dysregulation and neuroinflammation among others (15).

Therefore, the relationship between plasma p-tau181 levels and MBI supports its value as an early clinical marker of underlying tauopathy in the context of AD. In order to better understand the temporal relationship between plasma Aβ42/Aβ40, p-tau181 and MBI, longitudinal studies with larger sample sizes using both types of biomarkers are needed.

### MBI and blood-based Neurofilament light chain (NfL)

Neurofilament light chain (NfL) constitutes a neuronal cytoskeletal protein, playing a pivotal role in the maintenance of neuronal structure, stability and axonal transport (52). Under normal conditions, NfL is highly expressed and contained inside the neuronal axons, while during neuronal injury or degeneration, NfL is released extracellularly and can be detected in body fluids, such as the CSF or blood (53). Given its sensitivity to axonal damage, NfL has emerged as a valuable marker of early neurodegeneration and progression of several neurological diseases, including AD, Parkinson’s disease (PD) and atypical parkinsonian syndromes (APS), amyotrophic lateral sclerosis (ALS), and multiple sclerosis (MS) among others (52, 53). NfL is considered a general marker of neurodegeneration, and although it is not AD-specific, higher levels of NfL have been correlated with more rapid cognitive decline and disease progression (52). In terms of the A/T/N framework, NfL can reflect the “N” of neurodegeneration (54).

Although NfL has been initially investigated in the CSF, a growing body of evidence supports their utility as blood-based biomarkers. Recent advances in the detection of NfL through assay technologies including the ultra-sensitive single-molecule array (SIMOA) assays have enabled the reliable measurement of NfL in blood samples. Importantly, plasma NfL levels have also been associated with cognitive status and other well-established markers of AD-related neurodegeneration (52). More specifically, a longitudinal study in patients with MCI and AD dementia demonstrated that increased plasma NfL levels over time were correlated with poorer global cognitive performance and AD biomarkers in the CSF at baseline (low amyloid beta 42, high total tau and high phosphorylated tau levels) (43). Another study in non-demented individuals indicated that higher CSF and plasma NfL levels at baseline were correlated with longitudinally lower cognitive performance (55). Notably, NfL alterations in the CSF, plasma and serum have been detected prior to the clinical onset of cognitive complaints, highlighting their sensitivity as an early indicator of the underlying neurodegenerative process (20, 56). However, the relationship between NfL levels and NPS in the context of MBI was until recently largely unexplored.

In this regard, a recent study by Naude et al. (20) based on the data from Alzheimer’s Disease Neuroimaging Initiative (ADNI) aimed to investigate the association between MBI and the longitudinal change of blood NfL levels in 584 non-demented older individuals. In this study, the presence of MBI at baseline, as assessed by the NPI-Q, was associated with a more rapid increase in NfL concentration over a 2-year period (20). Notably, MBI was the only variable that could significantly predict the change in NfL levels, after controlling for cognitive status (normal cognition versus MCI), age, sex and years of education (20). In line with this evidence, no significant differences have been found in the rate of NfL changes in another study when the stratification was based only on the cognitive status (normal cognition versus MCI) (43). In agreement with the abovementioned evidence, higher plasma NfL levels have been correlated with a longitudinal increase in the severity of NPS as assessed by NPI-Q in non-demented individuals (57). Collectively, this evidence further supports the hypothesis that MBI is an early, sensitive marker of neurodegeneration, even irrespective of cognitive performance.

Furthermore, in the study by Naude et al. (20), participants that developed NPS at the follow-up visit at 2 years demonstrated higher NfL levels at baseline, compared to those who remained without NPS during this period (20). It could be speculated that NfL accumulation, reflecting the axonal degeneration, might precede the clinical onset of NPS in the context of MBI (20). This consideration argues that MBI has a neurobiological basis and is an integral part of the disease, as opposed to the perspective of “reverse causality,” according to which MBI itself causes cognitive impairment.

Therefore, it can be proposed that beyond the neurocognitive axis of pre-dementia states, NfL changes might also reflect the trajectory of the neurobehavioral axis represented by the NPS in the context of MBI. MBI may also indicate faster disease progression, revealed by the greater NfL increase over time. These results aid in confirming the validity of MBI and highlight its importance as an early marker of not only the presence but also the rate of progression of neurodegeneration.

### Other emerging potential blood-based biomarkers

#### Homocysteine

Besides the evidence linking MBI with plasma p-tau181 in the study by Gonzalez-Bautista and colleagues, MBI and specific MBI domains were linked to dysregulated metabolic biomarkers (19). In particular, abnormal perception or thought content was correlated with a faster increase in plasma homocysteine levels (19), suggesting that dysregulation of homocysteine metabolism might contribute to the pathophysiology of MBI domain of abnormal perception or thought content.

Homocysteine constitutes an amino acid being critically implicated in DNA methylation, protein synthesis and other cellular functions (58). Trans-sulfuration to taurine and cysteine, as well as remethylation to methionine are the main pathways implicated in the metabolism of homocysteine, while vitamin B12, B6 and folate availability plays a key role in this process (58). Several enzymes are involved in homocysteine metabolism, including methionine synthase, methionine synthase reductase (MTRR), and the methylenetetrahydrofolate reductase (MTHFR) (58). MTHFR common polymorphisms, including the C677T polymorphism that is present in about 10–15% of the general population, are well-established risk factors for cardiovascular diseases (59).

Increased homocysteine levels have been related to cognitive impairment and psychiatric disorders, including schizophrenia (60). Elevated plasma total homocysteine is considered an important modifiable risk factor for cognitive decline, including AD dementia (61). In non-demented elderly individuals, higher homocysteine levels have been correlated with episodic memory deficits (62). In addition, elevated concentration of homocysteine has been inversely associated with global cognitive performance and hippocampal volume in non-demented individuals (63). Compared to cognitively normal individuals, patients with several dementia subtypes, including AD, vascular dementia, frontotemporal dementia and Lewy body dementia displayed elevated homocysteine levels (64). In patients with AD, increased homocysteine was associated with more pronounced medial temporal lobe atrophy (64). In a recent study, elevated homocysteine levels could increase the risk for conversion to dementia among patients with non-amnestic MCI (65), suggesting that homocysteine might represent a useful blood-based biomarker for incident cognitive decline.

In regard to psychiatric disorders, increased homocysteine levels have been reported in individuals with drug-naïve first-episode psychosis (66), as well as patients with chronic schizophrenia (67). Importantly, more severe negative symptoms in patients with schizophrenia have been correlated with higher homocysteine levels (68, 69). Although the neurobiology of primary psychiatric disorders, such as schizophrenia, differs from the MBI and dementia-related NPS, these findings imply that disrupted metabolism in homocysteine might pathophysiologically underlie, at least partially, the interconnection between psychosis and cognitive impairment.

Concerning the potential mechanisms, glutamatergic neurotransmission has been suggested to play an important role. Homocysteine and homocysteic acid, its metabolite, can act as agonists of the NMDA receptors, stimulating calcium influx, which contributes to neurotoxicity (60). Furthermore, animal studies have shown that hyperhomocysteinemia is related to lower serotonin and dopamine levels in the brain (70) and might dysregulate hippocampal synaptic plasticity (71). Oxidative stress, impaired energy metabolism, disrupted long-term potentiation, vascular injury, neuronal apoptosis as well as epigenetic mechanisms via abnormal DNA methylation have been also implicated in the neurotoxic properties of homocysteine (60), which might also underlie its effects in the NPS related to cognitive impairment. It has also been proposed that elevated homocysteine levels may stimulate tau hyperphosphorylation via modulating the activity of protein phosphatase 2A (PP2A) and glycogen synthase kinase-3 beta (GSK3β) (72), thereby contributing to neurodegeneration (19). Furthermore, the A1298C variant of the (*MTHFR*) gene might also play a significant role (19).

Hence, MBI and abnormal perception or thought content in particular might be linked to higher homocysteine levels. However, further evidence is needed before the use of this information in clinical practice.

#### Insulin and glucose metabolism related biomarkers

Besides the evidence on homocysteine, in the study by Gonzalez-Bautista and colleagues, impulse dyscontrol and decreased motivation were associated with faster reduction in the plasma levels of insulin during the 1-year of follow up (19), highlighting the potential role of impaired glucose metabolism and brain insulin resistance in the neurobiology of MBI. In accordance, in a recent study in Singapore, elevated fasting glucose levels were significantly associated with the MBI domain of social inappropriateness in non-demented individuals (73).

AD is considered as “type 3 diabetes,” and type 2 diabetes is a well-known risk factor for dementia (74, 75). A growing body of evidence shows that dysregulation of insulin-related pathways plays a significant role in the pathophysiology of AD (75). Dysfunction of the brain endothelium, excessive neuroinflammation, disruption of the blood–brain barrier and mitochondrial dysfunction have been suggested as possible mechanisms linking insulin resistance to neurodegenerative diseases (75). Of note, abnormal glucose metabolism is evident more than a decade before the clinical onset of AD (76), suggesting its implication in the early preclinical stages. In addition, longer disease duration and poorer glycemic control have been associated with faster cognitive decline in middle-aged patients with type 2 diabetes (77). Furthermore, increased insulin resistance has been linked to elevated amyloid deposition in frontal and temporal brain regions, as assessed by amyloid PET, in late middle-aged non-demented individuals with normoglycemia (78). Hence, it can be speculated that aberrant glucose glycose in glucose metabolism might contribute to both the development and progression of cognitive impairment in AD.

Type 2 diabetes has also been associated with psychiatric symptomatology, as poorer glycemic control may increase the risk of depression in diabetic patients (79). Interestingly, type 2 diabetes has been associated with a higher risk of NPS in patients with early stages of AD. In particular, anxiety, depressive symptoms and nighttime behavioral symptoms were significantly more common in diabetic individuals with early AD compared to those without diabetes (80). Inadequate use of glucose due to insulin resistance may also contribute to neuronal damage and subsequent degeneration in the limbic system and other areas of the brain, leading to behavioral manifestations (81). Therefore, it can be proposed that insulin resistance and dysregulated glucose metabolism might contribute to the pathophysiology of NPS at the early stages of AD, highlighting also its role as a potential biomarker. However, further longitudinal studies using a more comprehensive definition of MBI and MBI domains are needed to confirm this hypothesis.

#### Ferritin

A significant interaction between affective dysregulation and CDR score regarding the association between MBI and ferritin levels has also been described in the study by Gonzalez-Bautista et al. (19). These results suggest that as cognitive decline progresses, the relationship between this MBI domain and impaired iron homeostasis might become more pronounced.

Ferritin is the major protein for iron storage in the human body (82). Iron plays a pivotal role in critical cellular processes, including energy metabolism and oxygen transport, and it acts as a cofactor for several enzymes implicated in the synthesis of myelin and neurotransmitters, mitochondrial and lysosomal function, dopamine metabolism among others (83). Iron dyshomeostasis in the brain is one of the core contributors in the pathophysiology of several neurodegenerative diseases including AD (83). Higher levels of iron deposition have been detected in the cerebellum and cortex of individuals with MCI, and glial accumulations of redox-active iron had a tendency to increase as cognitive impairment progressed (84). Furthermore, iron overload has been shown to upregulate the Aβ precursor protein (APP), thereby promoting amyloid plaque deposition, while it can also stimulate tau phosphorylation (85). Ferritin levels are also higher in the hippocampus and cortex of AD patients, potentially contributing to faster progression of AD neuropathology, by promoting oxidative damage and subsequently neuronal loss (86, 87). These results imply that iron imbalance and abnormal ferritin levels might be an early process during neurodegeneration, contributing also to the progression of the disease.

Besides neurodegenerative diseases, ferritin and iron dyshomeostasis have also been related to psychiatric conditions. In this regard, lower transferrin and ferritin levels have been associated with depressive symptomatology in older individuals (88), and increased iron deposition in the thalamus and putamen has been shown in patients with depression (89).

Hence, metabolic imbalance in iron pathways might contribute to the pathophysiology of MBI or underlie a faster progression to cognitive decline. Future studies will clarify whether this mechanism might contribute to MBI, and whether ferritin or transferrin could be useful as potential biomarkers in the context of NPS in preclinical AD stages.

## Discussion and future perspectives

In summary, the growing body of literature evidence suggests that MBI might be associated with lower Aβ42/Aβ40 ratio, increased p-tau181 levels, as well as higher NfL levels in blood, even irrespective to cognitive performance. Since these biomarkers may represent the “A” – standing for amyloid, “T” – standing for tau and “N” – standing for neurodegeneration part of the ATN classification of AD, it seems that MBI is related to all these three biomarker types, corresponding to the relative AD pathological changes. There is also some evidence linking MBI with some blood-based metabolic markers of insulin resistance and dysregulated glucose metabolism, homocysteine and ferritin. However, further evidence is needed to confirm these associations and their clinical utility in the daily practice.

Similar to the cognitive axis represented by SCD and MCI, MBI is considered to represent the behavioral axis of the pre-dementia stages. This hypothesis is further supported by the associations between MBI and biomarkers of amyloid pathology, tau pathology and neurodegeneration, as described above. Based on the above evidence, it can be suggested that MBI could be used as an early clinical “marker” or a “proxy” of an underlying neurodegenerative process, representing an at-risk state of cognitive decline. MBI might be a useful, non-invasive, inexpensive, and scalable tool for both clinicians and researchers to systematically identify individuals with behavioral changes linked to incident cognitive impairment. This approach could allow for close monitoring, potential early intervention, as well as enrichment of participant pools in clinical trials.

The combined use of MBI and blood-based biomarkers would also help general practitioners in the primary care settings to make the appropriate referrals to specialized memory clinics. This approach would provide important benefits to patients, medical doctors and healthcare system, by avoiding unnecessary referrals and diagnostic work-up procedures.

An important limitation of studies examining the clinical and biological correlates of MBI is the inconsistent application of strict MBI criteria for categorizing participants into MBI-positive or MBI-negative groups. A potential reason is that the definition of MBI is relatively new, and the criteria had not been incorporated into the design of relative studies exploring existing datasets. In this regard, according to the definition of MBI, NPS should emerge during later life, and not be attributed to a prior psychiatric disorder. In the studies by Naude et al. (20) and Miao et al. (17), some participants with severe psychiatric diseases like clinically significant psychosis or depression were excluded from ADNI, considering that these might represent a primary psychiatric disorder (17). However, a natural history of the NPS is rather needed for differentiating MBI from a primary psychiatric disease, in order to assess whether the symptoms are longstanding, recurrent or with a new onset. As a result, some participants with MBI might be lost in this study. In the study by Gonzalez-Bautista and colleagues, participants in the NOLAN study with a history of a major psychiatric disorder (major depression, bipolar disorder) were excluded (19). Future studies should more precisely clarify the nature of psychiatric symptoms in order to distinguish between primary psychiatric disorders and NPS in the context of MBI.

The tool for assessing MBI symptoms is also very important. The NPI-Q was used in four out of the five studies described herein (17–20). As abovementioned, an algorithm has been developed for transforming the NPI-Q items into the MBI domains. However, the NPI-Q was specifically developed for patients with dementia and is not intended to capture the subtle behavioral changes in individuals with normal cognition or MCI. The MBI-C is a more precise instrument, since it has been explicitly created for identifying MBI among community-dwelling functionally independent older individuals (13). Furthermore, the use of one-month reference period -as in the NPI-Q-, instead of the six-month period needed for the MBI definition, reduces specificity, since reactive or transient neuropsychiatric manifestations might be also included in the category of MBI. To address this issue, Ghahremani et al. (18) defined MBI with NPI or NPI-Q score > 0 in two consecutive visits. Another potential mitigation approach to increase specificity, as followed by Naude and colleagues, could be to classify participants as MBI-positive only those with NPI-Q > 1, rather than using the cutoff NPI-Q ≥ 1, as proposed in the published algorithm (20). However, this method may result in reduced sensitivity, since some MBI cases with NPI-Q = 1 would be excluded. As the authors mentioned, the inclusion of participants with more severe NPS as a compensatory approach for the shorter reference period would rather not identify the actual “MBI signal” (20). Hence, further studies using the MBI-C are needed to validate the role of plasma Aβ42/Aβ40 ratio, p-tau181 and NfL levels in MBI, as well as in the different MBI domains.

Nevertheless, even with the limitation of the imprecise MBI definition and assessment, the findings of the abovementioned studies are very important as first steps toward our better understanding of the clinical entity and neurobiology of MBI.

In addition, although the longitudinal evidence described above shed some light on the relative timing of each observation, such as the onset of MBI symptoms or the changes in NfL levels over time, the exact temporal relationships between MBI and NfL, amyloid and tau biomarkers remain to be explored.

The elucidation of the relationship between MBI and blood-based biomarkers holds a promising potential for novel therapeutic strategies in AD and related neurodegenerative diseases. First, the identification of older adults at early stages of neurodegeneration via Aβ42/Aβ40, p-tau181 and NfL would aid in targeted and personalized therapeutic interventions, according to the pathophysiological profile of each patient. In addition, clarifying the metabolic and inflammatory disturbances associated with MBI through biomarkers like homocysteine, insulin and ferritin may pave the way for adjunctive therapeutic approaches targeting these mechanisms.

Pharmacological targeting of amyloid or p-tau with antibodies represents a promising disease-modifying therapeutic approach against the early stages of AD-related neurodegeneration. In terms of MBI, these strategies would offer dual benefits, suppressing the amyloid- or tau-driven neuronal damage while possibly alleviating neuropsychiatric symptoms, such as agitation and irritability. In this context, lecanemab, an anti-amyloid beta monoclonal antibody recently approved for MCI and mild AD dementia (90), could hold a promising potential for changing the natural trajectory of MBI and its progression to AD dementia. Future studies with real-world evidence would help to clarify these effects.

Furthermore, given the possible relationship between MBI and homocysteine, it can be speculated that vitamin B supplements might lower the risk of cognitive decline in non-demented individuals with NPS. A recent meta-analysis based on 21 randomized controlled trials demonstrated that vitamin B supplement in elderly patients with MCI or normal cognition has a significantly positive effect on global cognitive performance (91). Future studies stratifying participants according to their MBI status would aid in clarifying whether patients with MBI would benefit the most from this intervention.

Concerning the glucose metabolism dysregulation, it has been demonstrated that intranasal insulin may improve verbal memory in patients with MCI and AD dementia (92). In addition, metformin use among patients with AD and type 2 diabetes has been associated with lower probability of anxiety and depressive symptoms (93). Thus, further research is needed to clarify the potential role of antidiabetic drugs in MBI and its associated cognitive decline.

Furthermore, it has been indicated that individuals with MCI with higher plasma activity of the enzyme BACE1 -which is implicated in the APP cleavage for the generation of amyloid beta- have an increased risk for progression to AD dementia in 3 years (94). Hence, BACE1 activity in plasma might represent a useful biomarker for AD progression, and its relationship with MBI could be also explored in the future.

Plasma p-tau217 levels have shown better performance compared to p-tau181 in distinguishing AD from other neurodegenerative diseases including frontotemporal lobar degeneration, as well as stronger correlation with signals in tau PET (95). Studies using CSF have demonstrated that p-tau217 may represent an earlier biomarker of AD neuropathology compared to p-tau181. Therefore, future studies should also focus on the association of p-tau217 with MBI, and its value in predicting cognitive decline in the context of NPS in preclinical populations (96).

Also, p-tau217 could be used for monitoring individuals with MBI for evaluating the risk for cognitive decline. In individuals with MCI, increasing plasma p-tau217 levels over time has been correlated to a higher risk of progression to AD dementia and worsening cognitive performance (97). In this context, repeated measurements of p-tau217 levels at appropriate time intervals might be useful in the dynamic risk-stratification of individuals with MBI.

Importantly, besides amyloid and tau pathologies, additional proteinopathies often co-exist in the brain of older individuals, including *α*-synuclein, TDP-43, non-AD tauopathies and vascular pathologies (98). Given that MBI is also associated with other causes of dementia, such as vascular dementia, FTD and Lewy body dementia (21), future studies investigating the link between MBI with potential blood-based biomarkers of these neuropathologies are needed.

Increased NfL levels in the CSF and plasma have been associated with various neurodegenerative diseases, including frontotemporal dementia, amyotrophic lateral sclerosis, corticobasal syndrome, multiple system atrophy, and progressive supranuclear palsy, as well as other neurological diseases like multiple sclerosis, stroke and traumatic brain injury (10). NfL is not specific for AD, but rather a sensitive marker of axonal damage. Since MBI is also associated with other causes of dementia (21), it can be proposed that plasma NfL could be used as an initial screening tool among patients with MBI in primary care settings, as an indicator of neurodegeneration. As a second step, additional testing including plasma Aβ42/Aβ40 and p-tau181 could be performed for identifying the potential underlying neuropathology.

Neuroinflammation, characterized by the activation of glial cells (mainly astrocytes and microglia), the release of pro-inflammatory cytokines and subsequent neuronal damage, is highly implicated in the pathophysiology of AD and other neurodegenerative diseases (99). Emerging evidence highlights that abnormal neuroinflammatory responses are present even at the earliest AD stages, before the clinical onset of cognitive decline (99). Several blood-based neuroinflammatory biomarkers have been investigated in AD, including glial fibrillary acidic protein (GFAP), a marker of astrocytic reactivity, soluble Triggering Receptor Expressed on Myeloid Cells 2 (sTREM2), related to microglial activation, as well as chitinase-3-like protein 1, also known as YKL-40 (99). Notably, a recent longitudinal observational study demonstrated that during the preclinical stage of AD, higher GFAP blood levels at baseline were associated with an increased risk of progression from normal cognition to MCI (100). The only study that investigated the relationship between inflammatory markers (GFAP) and MBI found no relative statistically significant associations (19). However, given the significant role of neuroinflammation in preclinical stages of AD, further longitudinal studies with larger sample sizes are needed in order to clarify this relationship.

Given the growing number of potential biomarkers of AD and the increasing literature evidence, future studies should focus on identifying the optimal multimodal combination marker for predicting AD, which could also enable biomarker-guided treatment and preventive approaches. A combined investigation of several biomarkers that reflect diverse pathophysiological mechanisms (amyloidopathy, tauopathy, metabolic disturbances, neuroinflammation etc.) would also enable the elucidation of the underlying mechanisms of neurodegeneration.

## Conclusion

The behavioral changes captured by MBI combined with the neurobiological validation that is provided by the amyloid, tau and NfL biomarkers may provide a sensitive approach for detecting and monitoring the individuals at risk for cognitive decline, even irrespective of their cognitive performance. The association between MBI and biomarkers of neurodegeneration reinforces the hypothesis that MBI may be a core, early marker of AD or other neurodegenerative diseases related to cognitive impairment. In addition, MBI symptoms might be an indicator of an accelerated neurodegenerative process, possibly resulting in more rapid clinical progression.

MBI assessment via the MBI-C represents a non-invasive, scalable tool for evaluating the NPS in pre-dementia at-risk states. Individuals with MBI could subsequently undergo a detailed clinical assessment, appropriate work-up, and subsequently further risk stratification based on blood or other biomarkers. This population can also be considered for clinical trials related to dementia, particularly those focusing on primary or secondary prevention strategies. In this way, MBI assessment might serve as an efficient and inexpensive additional screening tool for clinical trials, resulting in increased study power and lower cost (20).

Collectively, future evidence on the neurobiology, biomarkers and clinical implication of MBI will help toward the development and assessment of the efficacy of novel prevention and treatment approaches early during neurodegeneration.
